# The ultrastructural characteristics of porcine hepatocytes donated after cardiac death and preserved with warm machine perfusion preservation

**DOI:** 10.1371/journal.pone.0186352

**Published:** 2017-10-12

**Authors:** Hiroki Bochimoto, Naoto Matsuno, Yo Ishihara, Tatsuya Shonaka, Daisuke Koga, Yoshiki Hira, Yuji Nishikawa, Hiroyuki Furukawa, Tsuyoshi Watanabe

**Affiliations:** 1 Health Care Administration Center, Obihiro University of Agriculture and Veterinary Medicine, Obihiro, Hokkaido, Japan; 2 Department of Surgery, Asahikawa Medical University, Asahikawa, Hokkaido, Japan; 3 Department of Microscopic Anatomy and Cell Biology, Asahikawa Medical University, Asahikawa, Hokkaido, Japan; 4 Area of Functional Anatomy, Department of Nursing, Asahikawa Medical University, Asahikawa, Hokkaido, Japan; 5 Department of Pathology, Asahikawa Medical University, Asahikawa, Hokkaido, Japan; Virginia Commonwealth University, UNITED STATES

## Abstract

The effects of warm machine perfusion preservation of liver grafts donated after cardiac death on the intracellular three-dimensional ultrastructure of the organelles in hepatocytes remain unclear. Here we analyzed comparatively the ultrastructure of the endomembrane systems in porcine hepatocytes under warm ischemia and successive hypothermic and midthermic machine perfusion preservation, a type of the warm machine perfusion. Porcine liver grafts which had a warm ischemia time of 60 minutes were perfused for 4 hours with modified University of Wisconsin gluconate solution. Group A grafts were preserved with hypothermic machine perfusion preservation at 8°C constantly for 4 hours. Group B grafts were preserved with rewarming up to 22°C by warm machine perfusion preservation for 4 hours. An analysis of hepatocytes after 60 minutes of warm ischemia by scanning electron microscope revealed the appearance of abnormal vacuoles and invagination of mitochondria. In the hepatocytes preserved by subsequent hypothermic machine perfusion preservation, strongly swollen mitochondria were observed. In contrast, the warm machine perfusion preservation could preserve the functional appearance of mitochondria in hepatocytes. Furthermore, abundant vacuoles and membranous structures sequestrating cellular organelles like autophagic vacuoles were frequently observed in hepatocytes after warm machine perfusion preservation. In conclusion, the ultrastructure of the endomembrane systems in the hepatocytes of liver grafts changed in accordance with the temperature conditions of machine perfusion preservation. In addition, temperature condition of the machine perfusion preservation may also affect the condition of the hepatic graft attributed to autophagy systems, and consequently alleviate the damage of the hepatocytes.

## Introduction

The shortage of brain-dead donor liver grafts is a serious problem worldwide. One way of expanding the donor organ pool is by using grafts donated after cardiac death (DCD). However, the use of DCD liver grafts incurs a higher risk of primary nonfunction or ischemia-reperfusion injury. The superiority of machine perfusion preservation (MP) to simple cold storage was recently reported in clinical kidney preservation [1,2]. Similarly, in the field of liver transplantation, strategies as MP with oxygen and nutrition-containing solution have the potential to improve the outcome of liver transplantation with marginal grafts by reducing preservation injury and improving graft assessment [3,4]. MP of DCD liver grafts are roughly categorized into two groups: cold MP and warm MP (WMP) [3,5,6]. Many studies have shown that the cold MP, named hypothermic MP (HMP) first introduced in accordance with preceding MP of kidney [7], improved graft function and attenuated classical biochemical markers of liver preservation injury compared to simple cold storage [8–16]. In addition, WMP had emerged as a novel strategy, which maintain liver grafts at a more physiologic temperature compared to HMP to avoids cold ischemic injury and offers the opportunity to assess and possibly repair a metabolically active liver graft [3,6,17,18]. WMP, including 3 subcategories by temperature range [19]: midthermic MP (MMP, 13-24C), subnormothermic MP (SMP, 25-34C) and normothermic MP (NMP, 35-38C), have already proven advantageous in reducing markers of biliary injury during preservation and restoring normal biliary physiology [20]. Furthermore, WMP of DCD liver grafts have demonstrated the good result for the graft function and transplantation in rat, porcine and human [20–37]. Matsuno et al. directly showed that the AST and LDH levels in the effluent were lower in MMP or SMP (22-25C) with gradual rewarming as a type of WMP compared with HMP after temporal hypothermia subsequent warm ischemia [38–40]. Matsuno et al. subsequently described that the utility of MMP or SMP with gradual rewarming are more relevant clinically than HMP under the reality situation of clinical organ retrieval requiring a period of cold preservation due to transport between institutions [39,41].

However, the effects of each type of MP on the intracellular ultrastructure of organelles, including the mitochondria in hepatocytes, differ, and few studies have examined these effects in detail. A number of reports have described the ultrastructural changes in hepatocytes under conditions of warm ischemia or various types of MP using a transmission electron microscope (TEM) [42–45]. However, three-dimensional (3D) intracellular ultrastructure, particularly the shape of the mitochondrial cristae, has been unclear, partly due to the limitations inherent in the two-dimensional images obtained by a TEM [46,47]. This dimensional limitation may be resolved by using osmium maceration, in which the specimens are immersed in a diluted osmium tetroxide solution to remove the cytoplasmic matrix [48]. Osmium maceration enables the clarification of the 3D ultrastructure of organelles composed of lipid components.

We recently found osmium maceration useful for determining the 3D ultrastructure in porcine hepatocytes [49]. Subsequently, in this study, we comparatively investigated the changes in the ultrastructure of the endomembranous systems, including the mitochondria, endoplasmic reticulum, and autophagosomes in porcine hepatocytes under warm ischemia and successive HMP or WMP. Based on this, we also discussed the putative theories of WMP as more optimal conditions of preservation of DCD liver grafts.

## Materials and methods

### Antibodies

Mouse monoclonal anti-cytochrome C antibodies were purchased from Promega Corporation (Madison, WI, USA; product code G7421). Rabbit polyclonal anti-LC3 antibodies were purchased from Medical and Biological Laboratories (Nagoya, Japan; product code PM036). Secondary antibodies conjugated with fluorescent dyes (Alexa Fluor 488- and 594-conjugated donkey polyclonal anti-rabbit- and anti-mouse-IgGs) were purchased from Invitrogen (Carlsbad, CA, USA) for observation with a confocal laser microscope.

### Animals

Domestic female cross-bred Large-Yorkshire, Landrace, and Duroc pigs (approximately 25 kg, 2 to 3 months old) were purchased from Daisetsusanrokusya Co., Ltd. All animal work was conducted according to the Guide for the Care and Use of Laboratory Animals at Asahikawa Medical University. All animal studies and procedures were approved by Asahikawa Medical University Animal Research Committee (permit no. 14172).

### Perfusion preservation machine

**Fig 1:**
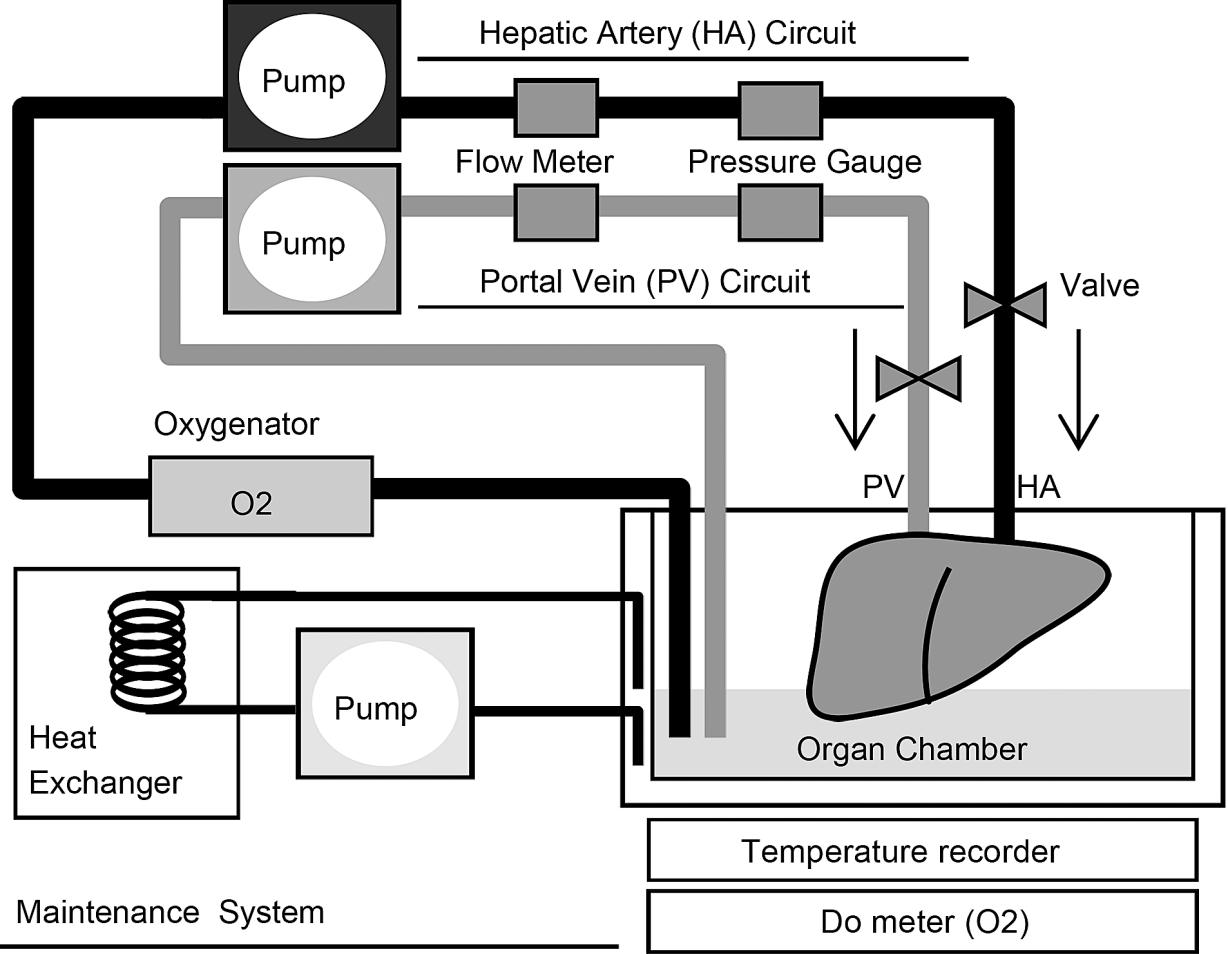
Schematic representation of the continuous machine perfusion system.

Porcine livers were perfused with a machine perfusion system (Fig 1) described previously [39]. The system consists of 2 separate circulating perfusion circuits for the portal vein (PV) and hepatic artery (HA), each has a roller pump, a flow meter and a pressure sensor, allowing nonpulsatile and pulsatile flow, respectively. An oxygenator was installed in the HA circuit. Both circuits were connected via plastic connectors to the hepatic vessels. Waterproof thermocouples installed in this system measured the solution and the organ temperatures. Furthermore, a dissolved oxygen (DO) meter was installed in this system to measure the DO concentration of solution. A computer records data and controls flow conditions and temperatures of the preservation solution. The temperature in the organ chamber was controlled by a heat exchanger and ice-cold water. As described previously [38–41], the flow rate was controlled as 0.22 and 0.06 mL/min per gram for the PV and HA, respectively.

### Preparation and preservation of the DCD liver

Six pigs weighing approximately 25 kg each were used as donors. In the present research, under inhalation anesthesia with isoflurane (Forane, Abbott, Japan), the pigs were laparotomized and the tissue samples of liver were biopsied from at least three distinct regions as control. Then, cardiac arrest was induced by intravenous injection of potassium chloride described previously [38][49], and removal of ventilation. The induction of cardiac arrest set as the starting point of warm ischemia. During warm ischemia, we performed the peeling around the blood vessels including portal vein and hepatic artery to connect with organ flush lines. Sixty minute after warm ischemia, the tissue samples of liver biopsied from at least three distinct regions, and the liver was procured to initially flushed with Euro-Collins solution via portal vein and hepatic artery at 8°C as a back table operation. Immediately, the organ flush lines connected to the perfusion preservation machine and the livers were perfused via both the hepatic artery and the portal vein in a closed circuit. Livers were perfused for 4 h with modified University of Wisconsin gluconate solution. The temperature conditions of the machine perfusion were divided to two groups, A and B. In Group A (n = 3), grafts were perfused at 8°C constantly as HMP. In Group B (n = 3), grafts were gradually rewarmed from 8 to 22°C during perfusion as MMP. These experimental conditions of groups A and B correspond to the groups of “2” (HMP after warm ischemia) and “3” (MP with rewarming after warm ischemia) in the previous study [38], respectively. Four hours after each MP, the tissue samples of liver were collected from at least three distinct regions of well-perfused area of a liver graft. All the samples were immediately treated with each fixative as described below for the analysis.

Hepatocellular damages of the preserved livers were evaluated by aspartate aminotransferase (AST), lactate dehydrogenase (LDH) levels in perfusate as described previously [38]. Perfusate was collected from the suprahepatic vena cava at 4 hours after reperfusion of each MP.

### Transmission electron microscopy

For observation by TEM, the samples biopsied from the liver of the experimental animals were cut into small pieces and immediately transferred into a fixative mixture of 2% glutaraldehyde /2% paraformaldehyde (PFA) in 0.1 M phosphate buffer (PB, pH adjusted to 7.4) for 2 h at 4°C. After washing thoroughly with 0.1 M PB containing 7.5% sucrose, the samples were further fixed with 1% osmium tetroxide (OsO4) in 0.1 M PB for 2 h at 4°C. The samples were then washed thoroughly with 0.1 M PB containing 7.5% sucrose, dehydrated in ascending concentrations of ethanol (50%, 70%, 95%, and 100%), transferred into propylene oxide, infiltrated, and embedded in epoxy resin (Epon 812). The ultrathin sections from the samples embedded in Epon 812 were then contrasted with saturated aqueous solutions of uranyl acetate and lead citrate, and examined using a TEM (H-7650; Hitachi High Technologies, Tokyo, Japan).

### Scanning electron microscopy

Tissue samples for observation by a scanning electron microscope (SEM) were prepared in accordance with the osmium maceration methods described previously [48]. Briefly, the samples biopsied from the liver of the experimental animals were cut into small pieces and immediately immersed into a fixative mixture of 0.5% PFA and 0.5% glutaraldehyde in 0.1 M PB (pH 7.4), for 30 min at 4°C. After fixation, the tissue blocks were directly immersed in 1% OsO4 in 0.1 M PB for 6 h at 4°C. The samples were then washed thoroughly with 0.1 M PB three times, immersed in 25% and 50% dimethyl sulfoxide for 30 min each for cryoprotection, and frozen on a metal plate deeply chilled with liquid nitrogen. The frozen liver blocks were cracked into 2 pieces with a screwdriver and a hammer and transferred into 50% dimethyl sulfoxide for thawing. After freeze cracking, the samples were washed 3 times in 0.1 M PB and then transferred to 0.1% OsO4 diluted with 0.1 M PB for 100 h at 20°C under light for osmium maceration. The macerated tissues were immersed in 1% OsO4 in 0.1 M PB for 1 h for further fixation. After rinsing with 0.1 M PB, the samples were treated with 1% tannic acid in 0.1 M PB and then with 1% OsO4 in 0.1 M PB for conductive staining. The conductive-stained samples were then dehydrated in ascending concentrations of ethanol (70%, 80%, 90%, 95%, and 100%), transferred into isoamyl acetate, and dried in a critical point dryer (HCP-2; Hitachi Koki Co., Ltd., Tokyo, Japan) using liquid CO_2_. The dried samples were mounted onto an aluminum metal plate and coated with platinum-palladium in an ion sputtering device (E1010; Hitachi Koki Co., Ltd.). After the process described above, the specimens were observed using a field emission SEM (S-4100; Hitachi High Technologies).

The relative frequency (RF) of abnormal mitochondria was calculated from SEM images with a magnification of ×10,000; representative 3 images were taken from each sample, and subsequently RF of abnormal mitochondria was calculated in each image. Mitochondrial area measurements were generated from SEM images with a magnification of ×10,000; representative images were taken from each sample of a minimum of 3 surfaces apart within the tissue block and hepatocyte mitochondria were analyzed. ImageJ software was used to calculate the area by drawing around of each individual mitochondrion analyzed. At least 300 measurements for each experimental group were generated.

### Immunofluorescence microscopy

For immunofluorescence microscopy, the samples biopsied from the liver of the experimental animals were cut into small pieces and immediately transferred into a fixative mixture of 4% PFA in 0.1 M PB (pH 7.4) containing 4% sucrose for 2 h at 4°C. After washing thoroughly with 0.1 M PB containing 7.5% sucrose, the samples were immersed sequentially in 15% sucrose (for 6 h) and 30% sucrose (for 12 h) solutions buffered in 0.1 M PB at 4°C, and then the tissue blocks were frozen at –30°C in the Tissue-Tek O.C.T. compound (Sakura Finetek, Tokyo, Japan). Tissue sections of 15 μm thickness were cut from the frozen tissue blocks with a cryostat (Leica Microsystems GmbH, Wetzlar, Germany) and mounted on microscope glass slides. These sections were treated with a 0.05% citraconic anhydride solution (Immunosaver; Nissin EM Co., Ltd., Tokyo, Japan) for 30 min at 60°C for antigen retrieval [50]. The tissue sections were permeabilized with a 0.2% Triton X-100 solution for 10 min at 20°C and then incubated with 2% normal donkey serum (30 min, 20°C) for blocking. After these pretreatments, tissue sections were incubated with a mixture of primary antibodies of different species (rabbit and mouse origin) for 16 h at 20°C. The sections were subsequently incubated with a mixture of the appropriate sets of Alexa Fluor 488- and 594-labeled secondary antibodies for 3 h at 20°C. Between each step, the sections on the microscopic slides were washed 3 times with 0.01 M PB (pH 7.4) containing 0.5 M NaCl and 0.1% Tween 20. After nuclear counter staining with 4′-6-diamidino-2-phenylindole, the coverslips were then mounted onto the tissue sections in 90% glycerol (vol/vol in PBS) containing 0.1% p-phenylenediamine dihydrochloride (Sigma-Aldrich). The stained sections were viewed with a confocal laser microscope (FV-1000D; Olympus, Tokyo, Japan).

### Statistical analysis

Data in the text and figures represent the means ± SEM. Unpaired two-tailed t tests were used to compare the significance of differences between two groups.

## Results

### Observation of intracellular ultrastructure of hepatocytes by TEM and SEM

**Fig 2:**
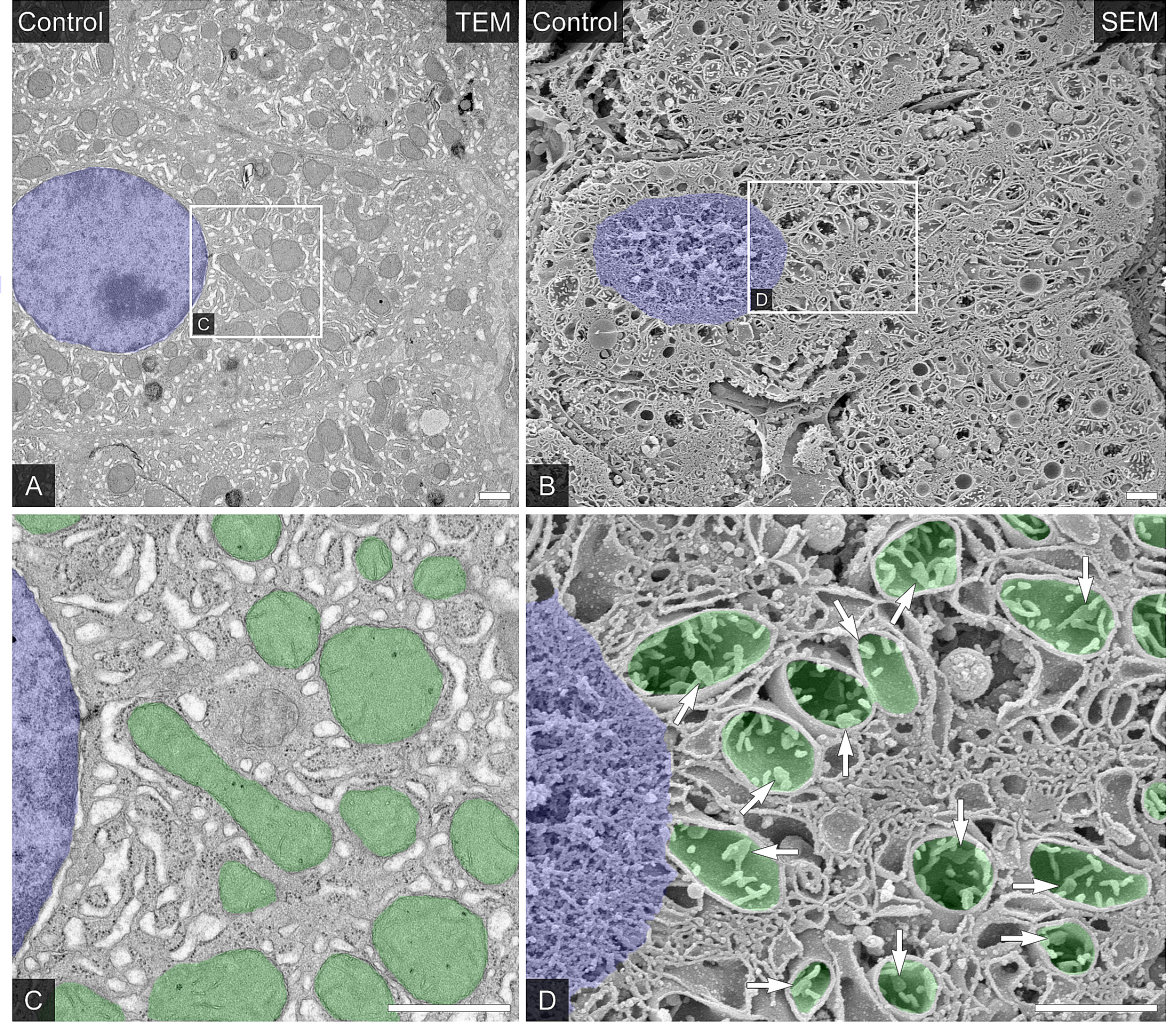
The ultrastructure of the endomembrane structure in the hepatocytes of the control liver. (A and C) Typical hepatocytes were identified in the ultrathin sections of the Epon 812-embedded control liver tissue (A). Nucleus was colored blue. The partial area indicated in A was further photographed at a higher magnification (C). Nucleus was colored blue and mitochondria were colored green. Bars = 1 μm. (B and D) Similar typical hepatocytes in osmium-macerated control porcine liver tissues were viewed with a scanning electron microscope (B). Nucleus was colored blue. The p area indicated in B was further photographed at a higher magnification (D). Nucleus was colored blue and mitochondria were colored green. Open arrows indicated cristae of mitochondria. Bars = 1 μm.

First, we examined the correspondence of the findings on TEM and SEM observation. At low magnification, TEM observation of hepatocytes in control liver revealed that they were basically mononuclear and possessed large numbers of oval or sausage-shaped mitochondria (Fig 2A). The hepatocytes had extensive endoplasmic reticulum in their overall cytoplasm, and their nuclear shape was a regular ellipse and their chromatin distributed uniformly (Fig 2A). The relationships between the cells appeared to be intact, as normal bile canaliculi with microvilli was formed by the plasma membrane of contiguous hepatocytes, which had a well-developed microvillus border. These hepatocytes and normal endothelial cells lined the space of Disse. These findings observed with a TEM corresponded relatively well to the low-magnification findings on observation with an SEM of the freeze-fractured surfaces of the control liver processed with osmium maceration. In addition, SEM observation clearly showed the details of the bile canaliculi, spaces of Disse, and intra-cisternal spaces of the membranous organelles, including some 3D conformation information (Fig 2B).

High-magnification observation with a TEM revealed that the nuclear envelope was clear and smooth, and reticulated-smooth endoplasmic reticulum was abundant in hepatocytes (Fig 2C). In addition, TEM observation showed that the mitochondria had normal inner and outer membranes and sparse cristae in the hepatocytes (Fig 2C, colored green). Additional observation with an SEM revealed that the well-developed tubular cristae connected mutually and occasionally (Fig 2D, open arrows). These data clearly showed well correspondence of observation of TEM and SEM. Furthermore, the SEM observation could demonstrate the 3D detail of intracellular organelles, which include the cristae of mitochondria in hepatocytes.

### Changes in the ultrastructure of organelles within hepatocytes after warming ischemia

**Fig 3:**
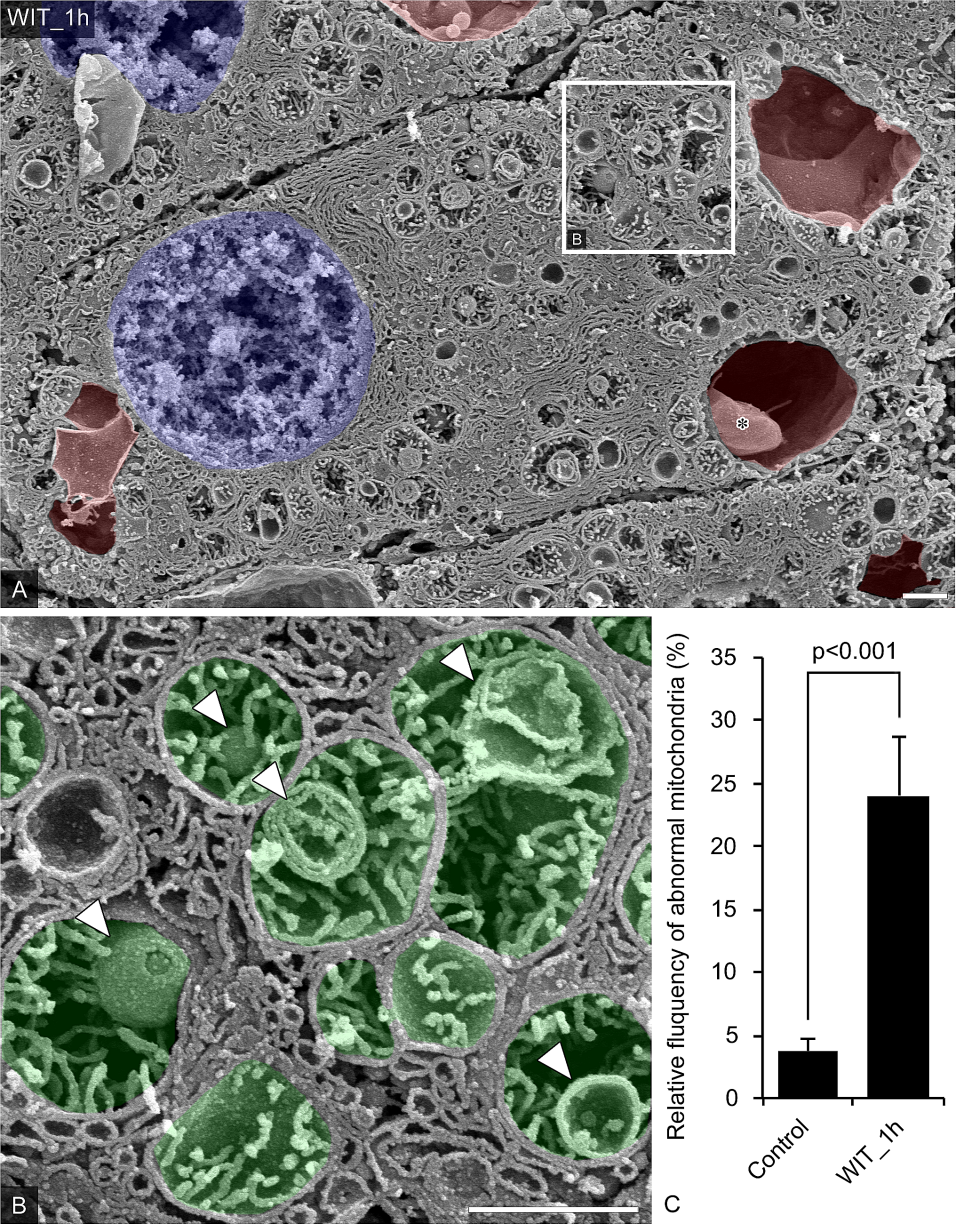
Changes in the ultrastructure of the endomembrane systems in porcine hepatocytes after warm ischemia. (A and B) Representative hepatocytes were observed by SEM in the osmium-macerated porcine liver graft samples after warm ischemia for 60 minutes. Nucleus was colored blue and abnormal vacuoles were colored red. Asterisks indicated the internal vacuole in an abnormal vacuole. The partial area indicated in A was further photographed at a higher magnification (B). Mitochondria were colored green. Intra-mitochondrial abnormal vesicles were indicated by open arrowheads. (C) Abnormal mitochondria was counted in SEM images (9–11 views of each groups, N = 3). Data represents as the means ± SEM. Unpaired two-tailed t-tests were used (p < 0.001). Bars = 1 μm.

After 1 h of warming ischemia, the color of the porcine liver samples had changed to dark red, macroscopically. Along with these changes in the appearance, the intra-cellular ultrastructure of hepatocytes was distorted. The low-magnification findings on observation with an SEM of the freeze-fractured surfaces of the liver after 1 h of warming ischemia processed with osmium maceration showed the contact between the hepatocytes had slightly loosened, both at the microvillus borders and at the junctions with sinusoidal endothelial cells. Generally, the endoplasmic reticulum increases in size and tends to have a lamellar morphology in hepatocytes (Fig 3A). However, the hepatic architecture and the relationships between the hepatocytes were largely maintained, as the cells formed normal bile canaliculi and interfaces. Noteworthy, the abnormal large vacuoles appeared in the hepatocytes (Fig 3A, colored red). These vacuoles were generally located near the nucleus but also appeared elsewhere in the cytoplasm. Occasionally, the large, abnormal vacuoles contained rather small vacuoles with a smooth surface (Fig 3A, asterisk). Observation of the freeze-fractured surface of these small vacuoles revealed a double membrane and the presence of small, empty vesicles (Panel A in S1 Fig). Interestingly, the internal space between the double membranes of a part of small vesicles was occasionally connected to the lysosome-like structure in cytoplasm (Panel A in S1 Fig, open arrowheads).

Even more interestingly, an SEM observation showed that the mitochondria in the hepatocytes were slightly swollen, with abnormal vesicles found in each mitochondrion. At high magnification, some intra-mitochondria vesicles had multi-lamellar membranes (Fig 3B, open arrowheads). Although these abnormal mitochondria were rarely observed in control, the RF was significantly increased in the liver after 1 h of warming ischemia (Fig 3C, control vs. WIT_1h, 3.75±0.93 vs. 24.05±4.59 percent per image, *P<0.001). Observation of other hepatocytes revealed that the outer membrane of the mitochondria had invaginated into the lumen with cytoplasm or an endoplasmic reticulum (Panel B in S1 Fig, open arrows).

### Comparison of changes in the intracellular ultrastructure with hypothermic and midthermic machine perfusion

**Fig 4:**
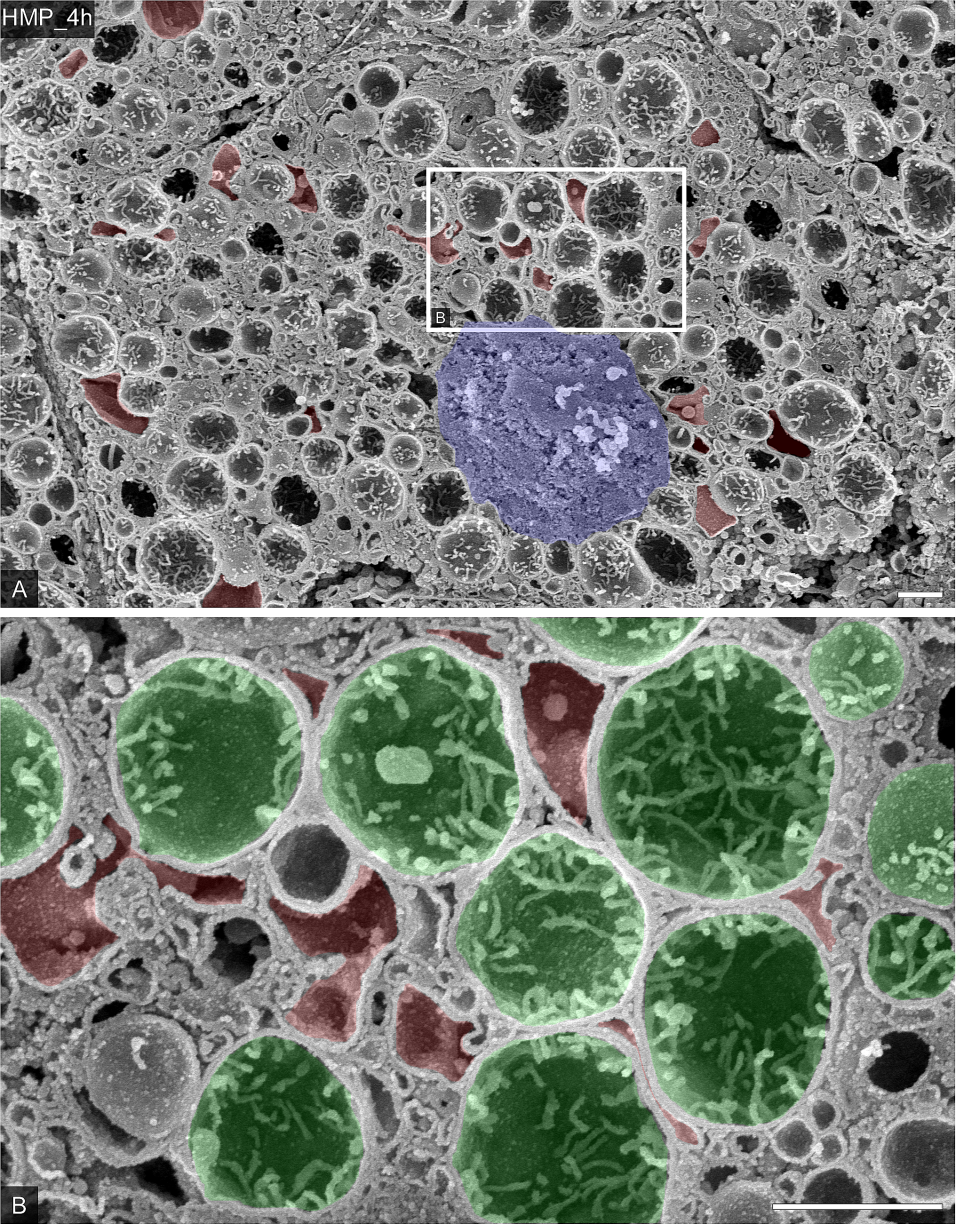
The ultrastructural alteration in porcine hepatocytes preserved by HMP. (A) Representative hepatocytes were observed by SEM in the osmium-macerated porcine liver graft samples preserved by HMP for 4 h after 60 minutes of warm ischemia. Nucleus was colored blue and abnormal vacuoles were colored red. The partial area indicated in A was further photographed at a higher magnification (B). Mitochondria were colored green and abnormal vacuoles were colored red. Bars = 1 μm.

The macroscopic appearance of the liver samples preserved with HMP (Group A) or MMP (Group B) similarly became pale after 4 h; however, the ultrastructure of the hepatocytes was significantly different between each condition followed the extent of hepatocellular damage in liver graft. As we reported previously [38], the value of lactate dehydrogenase (LDH) and aspartate aminotransferase (AST) in perfusate showed that the MMP significantly suppressed the increasing of the hepatic enzyme release compared to HMP (Panel A in S3 Fig. (LDH); HMP_4h vs. MMP_4h, 4760±856 vs. 1970±173 IU/L, Panel B in S3 Fig. (AST); HMP_4h vs. MMP_4h, 3170±250 vs. 2120±198 IU/L, *P<0.05, respectively). Correspondingly, observation by SEM revealed that the hepatic architecture and the connections between the hepatocytes were no longer visible, as the microvillus borders of the hepatocytes showed abnormal compaction and reduced volumes of canaliculi in the liver in Group A (Fig 4A).

**Fig 5:**
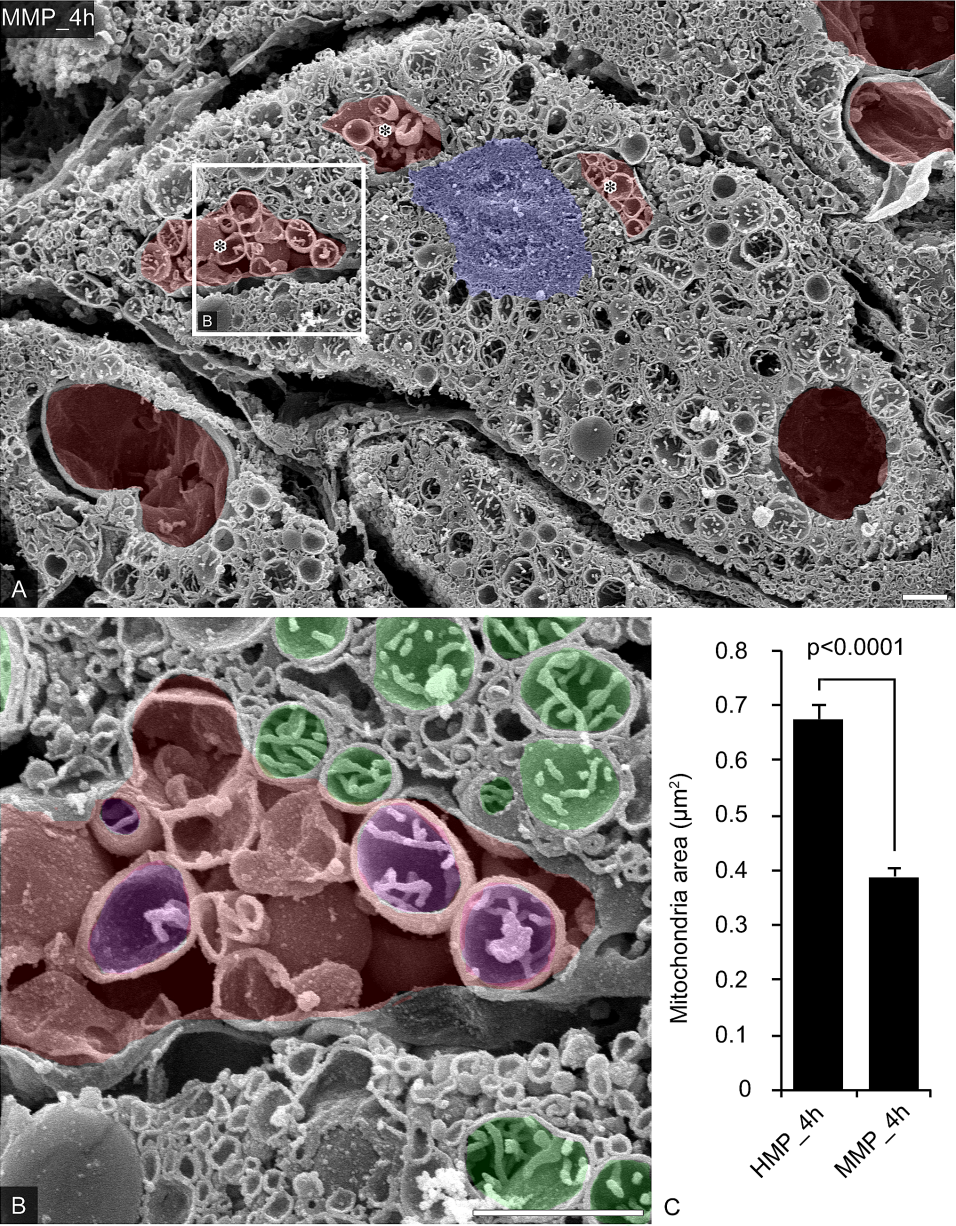
The ultrastructural characteristics in porcine hepatocytes preserved by MMP. (A) Representative hepatocytes were observed by SEM in the osmium-macerated porcine liver graft samples preserved by MMP for 4 h after 60 minutes of warm ischemia. Nucleus was colored blue and abnormal vacuoles were colored red. Asterisks indicated the vacuoles include the vesicles and mitochondria. The partial area indicated in A was further photographed at a higher magnification (B). Abnormal vacuoles were colored red. Cytoplasmic mitochondria were colored green and intravacuolar mitochondria were colored purple. (C) Mitochondria areas were measured in SEM images (9–11 views of each groups, N = 3). Data represents as the means ± SEM. Unpaired two-tailed t-tests were used (p < 0.0001). Bars = 1 am.

Furthermore, the presence of mitochondria in the cytoplasm and reticulated endoplasmic reticulum was observed in the hepatocytes in Group A (Fig 4A). At higher magnification, strongly swollen mitochondria and atrophy of cristae appeared in the hepatocytes in Group A (Fig 4B, colored green). Despite the disappearance of the large vacuoles observed at 60 min after warming ischemia, we did observe small vacuoles scattered around the cytoplasm in hepatocytes from Group A that wrapped the swollen mitochondria incompletely (Fig 4A and 4B, colored red). In contrast, in Group B, although the connections between the hepatocytes had loosened, the hepatocytes appeared more visible, as the canaliculi were of normal volume and the mitochondria appeared to have a normal function (Fig 5A). Interestingly, numerous abnormal large vacuoles remained in the cytoplasm of hepatocytes after 4 h of MMP (Fig 5A and 5B, colored red). Electron microscopy occasionally visualized abundant vacuoles and membranous structures sequestrating cellular organelles, just like the autophagosomes in hepatocytes (Fig 5A, asterisks). At high magnification, although the intravacuolar mitochondria were slightly swollen (Fig 5B, colored purple), cytoplasmic mitochondria rather maintained a functional small morphology and the cristae (Fig 5B, colored green). Mitochondrial swelling, generally reflected by an increase of the mitochondrial area is a well-accepted hallmark of dysfunction of this organelle [51,52]. Mitochondria area measurements in hepatocytes showed that the MMP significantly suppressed the increasing of mitochondria area compared to HMP (Fig 5C, HMP_4h vs. MMP_4h, 0.68±0.02 vs. 0.39±0.02 μm^2^ per mitochondria, *P<0.0001).

## Discussion

In this study we confirmed the usefulness of the SEM observation of osmium-macerated tissue samples for description of ultrastructure of hepatocellular organelles, especially the internal architecture of mitochondria (Fig 2). Based on this, we revealed the ultrastructural characteristics along with some 3D information of hepatocytes from porcine liver grafts after warm ischemia and subsequent MP after cardiac death by using SEM. Simple warm ischemia of liver grafts for 60 minutes affects the ultrastructural characteristics of hepatocytes.

One of the abnormal findings was the peculiar appearance of a multilamellar body in mitochondria after warm ischemia (Fig 3B). Similar structures in the hepatocytes of a murine ischemia reperfusion model were reported as autophagic vacuoles on TEM observation [53]. These autophagic vacuoles appeared under the condition of autophagy induction, as warm ischemia and ischemia-reperfusion induced autophagy in hepatocytes [54–56]. Our results revealed that the autophagic vacuoles in mitochondria were formed by invaginations into the matrix space of the mitochondrial outer membrane at the site of contact with the endoplasmic reticulum (Panel B in S1 Fig). This finding is consistent with the fact that autophagosomes are formed at the site of contact of mitochondria with the endoplasmic reticulum [57].

The process of mitochondrial autophagy, termed mitophagy, has several distinct variants. In mitophagy induced by starvation or hypoxia, mitochondria deformation typically occurs in coordination with the sequestration of mitophagosomes. By contrast, in mitophagy induced by the onset of mitochondrial permeability transition (MPT), mitochondrial deformation is not observed [58–60]. In addition, hepatocytes under conditions of hypoxic stress suppress the production of reactive oxygen species (ROS) by the degradation of mitochondria with mitophagy [55]. Therefore, the autophagic vacuoles of mitochondria in hepatocytes suffering warm ischemia might be associated with hypoxia-induced mitophagy. This hypothesis is supported by the fact that the number of autophagic vacuoles in the mitochondria decreases after oxygenation by machine perfusion, regardless of the temperature (Figs 4 and 5).

Autophagy occurs at low basal levels in virtually all cells that perform homeostatic functions, including the removal of damaged organelles such as mitochondria, and is involved in the recycling of denatured proteins and metabolic catabolites [61,62]. Damaged organelles participating in ROS generation, including mitochondria, are sequestrated and removed by the autophagic process, and autophagy plays a role in suppression of ROS generation and subsequent apoptosis [63]. Autophagy prevents hepatocyte apoptosis or necrosis under conditions of hypoxia-induced oxidative stress [54,56,64,65]. Considering these facts, it seems that the formation of mitochondrial autophagosomes in hepatocytes during warm ischemia suggested that the autophagy system plays a role in protecting hepatocytes from hypoxic injury.

Another finding was the appearance of huge single-membrane vacuoles in the hepatocytes (Fig 3A). There are a number of reports of the appearance of huge vacuoles in hepatocytes of animal models under hypoxia-induced oxidative stress after warm ischemia or cardiac death [45,66–70]. In addition, the extent of parenchymal vacuolation in warm ischemia liver grafts reflects the severity of hepatocellular damage [71]. On 3D observation using an SEM, we found that the huge vacuoles frequently contained smaller vacuoles within them (Panel A in S1 Fig). SEM observation of the freeze-fractured surface revealed that the intravacuolar vacuoles with double membranes contained vesicles. Furthermore, these intravacuolar vacuoles were connected to lysosome-like structures in the cytoplasm (Panel A in S1 Fig, open arrowheads). In addition, the appearance of LC3 accumulation corresponding to the vacuoles suggests a relationship between the vacuoles and autophagy (Panel A and B in S2 Fig). The etiology of vacuolation of hepatocytes has never been completely clarified, but the co-localization of cytochrome C with the accumulation of LC3 suggests that the vacuolation is due to mitochondrial autophagosomes (Panel B in S2 Fig). This hypothesis is consistent with the theory that autophagy mediates the cellular resistance of hepatocytes to the mitochondrial death pathway by blocking the activation of caspase-8 and subsequent cytoplasmic release of cytochrome C [72,73].

The alterations of the ultrastructural characteristics in hepatocytes caused by warm ischemia lead to various outcomes depending on the temperature conditions of subsequent MP. For the first example, swelling of mitochondria and dilation of endoplasmic reticulum in hepatocytes are more severe after HMP than MMP relatively. The swelling of mitochondria is induced by the onset of MPT, suggesting that the damage of mitochondria results in the production of ROS [74]. In addition, the dilation of the endoplasmic reticulum suggests that it’s under stress. These differences in the intracellular ultrastructural outcomes in hepatocytes between each MP type give an explanation for the results of our previous study showing that the AST and LDH levels in the effluent were lower in WMP compared with HMP [39,38].

Second, membrane structures enclosing some of the organelles were discerned in the hepatocytes after MMP for 4 h. These membrane structures comprised a single membrane, similar to the vacuoles that appeared after warm ischemia. TEM observation of post-mortem rat hepatocytes has also shown the presence of vacuoles surrounding organelles [70]. Considering these facts, it seems that the membrane structures enclosing some of the organelles is associated to the vacuoles appeared after warm ischemia. In addition, a comparison of the immunohistochemistry findings and SEM observations showed that the large accumulations of LC3 co-localized with cytochrome C in hepatocytes after MMP corresponded well with the membrane structures enclosing mitochondria (Panel D in S2 Fig). These findings suggest that the membrane structures isolating the organelles, including the mitochondria, are related to autophagy. Some studies have reported that the WMP prompts energy metabolism recovery [75,76]. In addition, controlled oxygenated rewarming, which is similar to MMP, resulted in significantly increased gene expression and protein levels of autophagy-related beclin-1 [77]. Therefore, it seems that the membrane structures isolating the organelles, including the mitochondria, are related to the induction of macroautophagy.

Third, we found that the endoplasmic reticulum membrane was frequently attached to the surface of the swollen mitochondria after HMP for 4 h (Fig 4B). Immunohistochemistry showed the presence of a number of small accumulations of LC3 co-localized with cytochrome C in the cytoplasm of these hepatocytes (Panel C in S2 Fig). Salas et al. found that hypothermia was a decisive element in the production of hepatic autophagy in rats [78,79]. In addition, the appearances of swollen mitochondria in the hepatocyte of mice under conditions of hypothermia have suggested that the damage to mitochondria is induced by the hypothermic condition itself [78]. Extended times of HMP may also be subject to particular disadvantages and limitations with regard to endoplasmic stress [80,81]. Therefore, it seems that the hypothermic conditions of HMP induce mitophagy without mitochondrial deformation, due to the onset of MPT [59]. The validity and relevance of this hypothesis need further confirmation in future studies.

There is a limitation to our study. The relatively high level of the LDH and AST in perfusate of MMP suggest that the considerable hepatocellular damage exist even in the more protective method, MMP in this study. The warm MP with rewarming is promising, as more and more researchers are publishing results preferring these temperature settings [82]. However it has not been sufficiently defined the rewarming velocity of liver grafts, the critical temperature the liver should reach, and the period of warm MP needed to adjust metabolic parameters [82]. Therefore, it is necessary that our morphological analysis should be done on the more protective modified WMP to clarify the protective mechanism of the MP on the liver graft in future.

In conclusion, the temperature conditions for WMP alleviate the damage of the hepatic graft via ultrastructural changes of mitochondria potentially associated with autophagy in hepatocytes. This alleviation potentially depends on the metabolically active scenario where organs are supplied with nutrients and oxygen to re-establish homeostasis facilitated by WMP. However, autophagy-associated hepatocyte death has been reported to trigger liver graft dysfunction, indicating that the effect of autophagy on hepatocytes is still controversial [16]. Further physiological studies are needed to clarify the detailed mechanisms of the ultrastructural changes and autophagy in hepatocytes under various temperature conditions of perfusion preservation of liver grafts for more appropriate preservation of liver grafts donated after cardiac death.

## Supporting information

S1 Fig The appearance of abnormal vacuoles and ultrastructural changes in the mitochondria in porcine hepatocytes after warm ischemia. (A) Representative abnormal vacuoles were observed in porcine hepatocytes using SEM after warm ischemia for 60 minutes. An abnormal vacuole was colored red. A lysosome-like structure was colored blue. Open arrowheads indicated the connection between the abnormal vacuole and lysosome-like structure. Simultaneously, the abnormal invagination of the mitochondrial outer membrane into the matrix space was also observed (B). Mitochondria were colored green. Open arrows indicate invaginations of mitochondria. Bars = 1 μm.
(TIF)

S2 Fig The changes in the intracellular distribution of LC3 and cytochrome C in porcine hepatocytes after warm ischemia and subsequent preservation by HMP or MMP. (A-D) Tissue sections (thickness: 15 μm) of the sample of porcine liver biopsied at the time of pre-DCD (A), after 60 minutes of warm ischemia (B), and 4 h after starting the preservation by HMP (C) or MMP (D) were simultaneously immunostained with rabbit polyclonal anti-LC3 (visualized with Alexa Fluor 488; green pseudocolor in A-D) and mouse monoclonal anti-cytochrome C (visualized with Alexa Fluor 594; red pseudocolor in A-D) antibodies. The cell nucleus was also stained with DAPI (Sigma-Aldrich) and viewed with a 405-nm laser source (blue). Bar = 10 μm.
(TIF)

S3 Fig The changes in the perfusate enzymes after warm ischemia and subsequent preservation by HMP or MMP. (A) Levels of lactate dehydrogenase (LDH), and (B) levels of aspartate aminotransferase (AST) in the perfusate at 4 hours after hypothermic and midthermic machine perfusion preservation. Data represents as the means ± SEM. Unpaired two-tailed t-tests were used (p<0.05).
(TIF)
