# Supplementary figures and images for: The ultrastructural characteristics of porcine hepatocytes donated after cardiac death and preserved with warm machine perfusion preservation
Source: PLoS One. 2017 Oct 12;12(10):e0186352. doi: 10.1371/journal.pone.0186352 (PMC5638504; doi:10.1371/journal.pone.0186352)

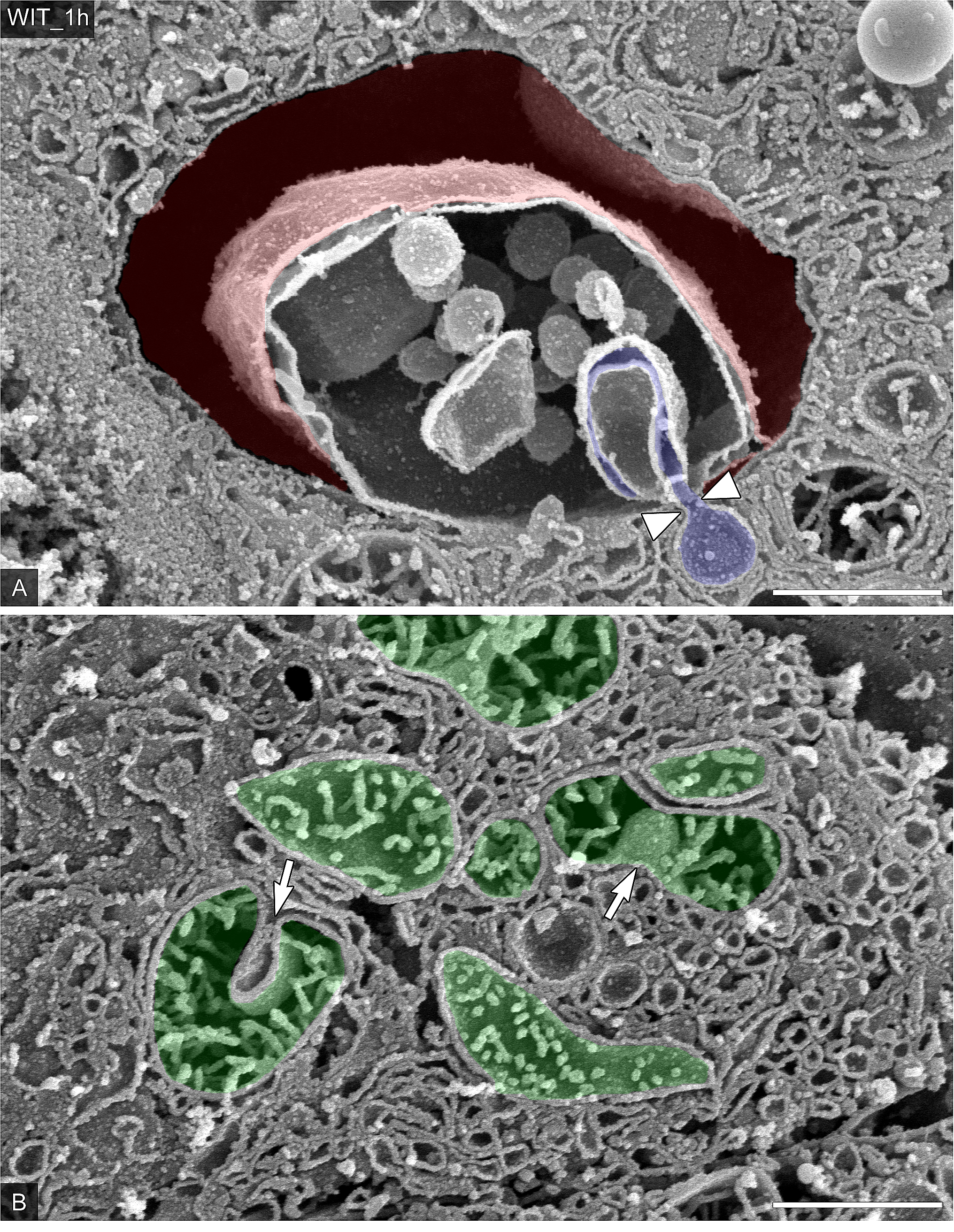

Supplement: S1 Fig — (A) Representative abnormal vacuoles were observed in porcine hepatocytes using SEM after warm ischemia for 60 minutes. An abnormal vacuole was colored red. A lysosome-like structure was colored blue. Open arrowheads indicated the connection between the abnormal vacuole and lysosome-like structure. Simultaneously, the abnormal invagination of the mitochondrial outer membrane into the matrix space was also observed (B). Mitochondria were colored green. Open arrows indicate invaginations of mitochondria. Bars = 1 μm. (TIF) [file pone.0186352.s001.tif]

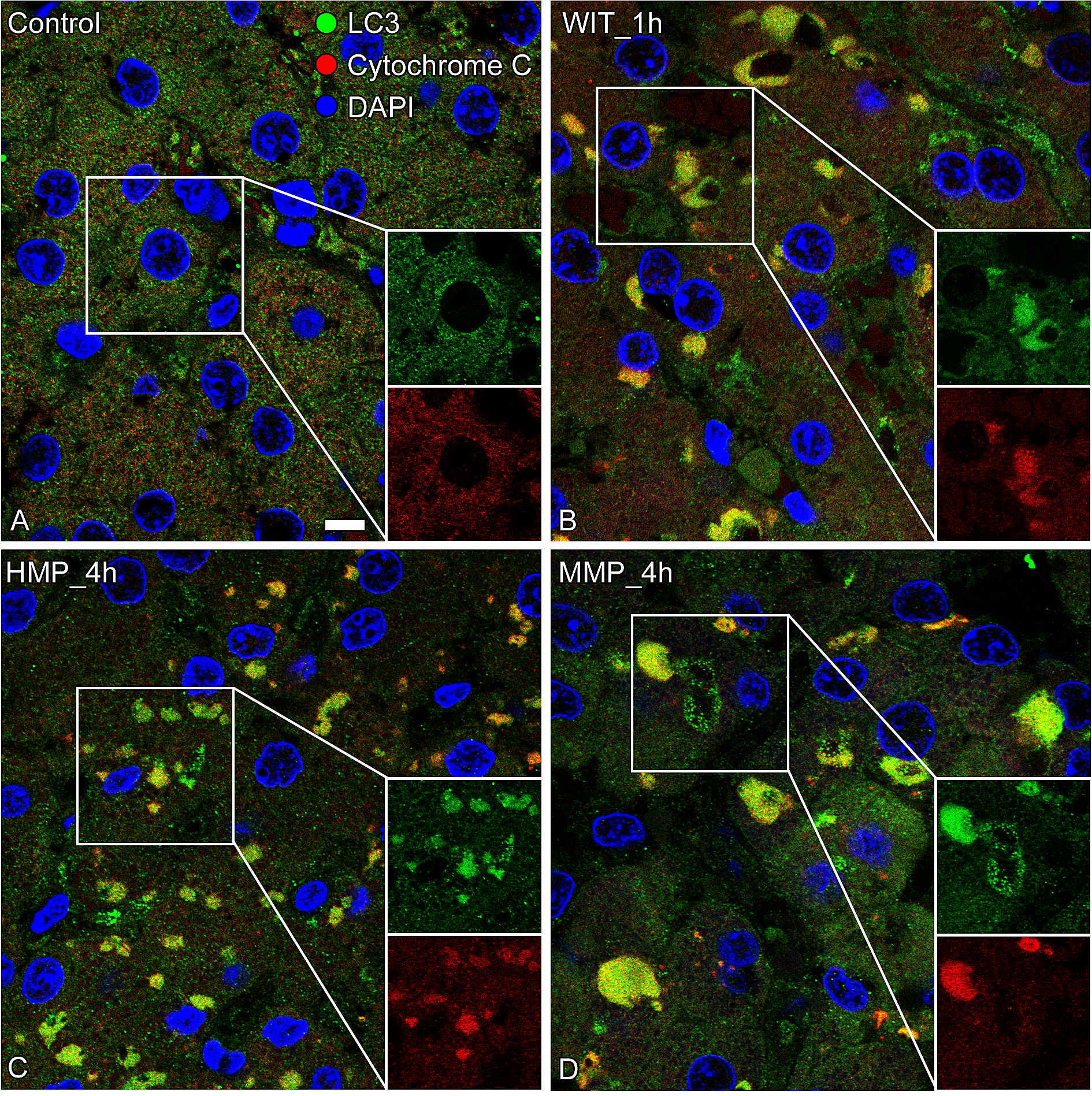

Supplement: S2 Fig — (A-D) Tissue sections (thickness: 15 μm) of the sample of porcine liver biopsied at the time of pre-DCD (A), after 60 minutes of warm ischemia (B), and 4 h after starting the preservation by HMP (C) or MMP (D) were simultaneously immunostained with rabbit polyclonal anti-LC3 (visualized with Alexa Fluor 488; green pseudocolor in A-D) and mouse monoclonal anti-cytochrome C (visualized with Alexa Fluor 594; red pseudocolor in A-D) antibodies. The cell nucleus was also stained with DAPI (Sigma-Aldrich) and viewed with a 405-nm laser source (blue). Bar = 10 μm. (TIF) [file pone.0186352.s002.tif]

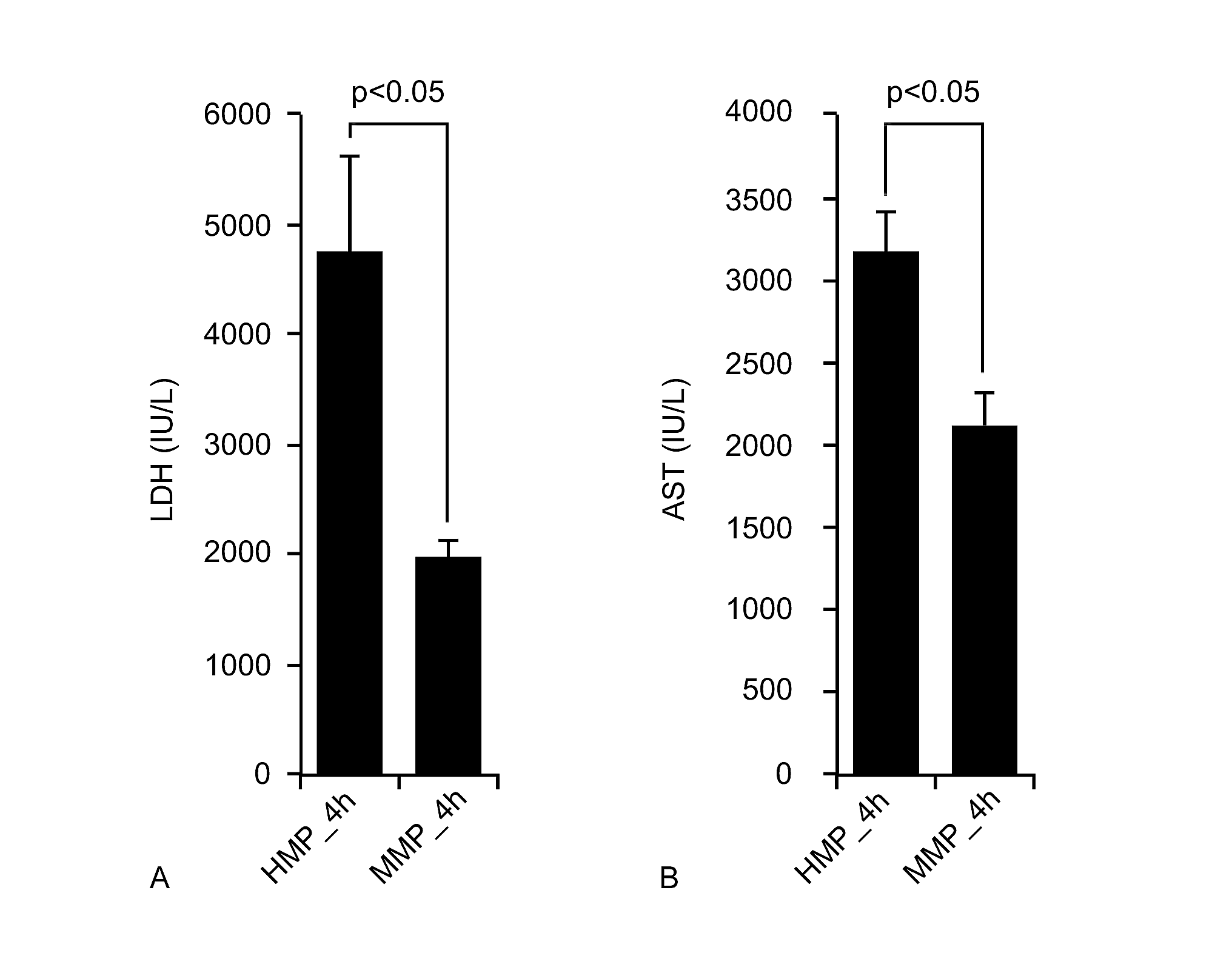

Supplement: S3 Fig — (A) Levels of lactate dehydrogenase (LDH), and (B) levels of aspartate aminotransferase (AST) in the perfusate at 4 hours after hypothermic and midthermic machine perfusion preservation. Data represents as the means ± SEM. Unpaired two-tailed t-tests were used (p<0.05). (TIF) [file pone.0186352.s003.tif]
